# Brain Development after Neonatal Intermittent Hyperoxia-Hypoxia in the Rat Studied by Longitudinal MRI and Immunohistochemistry

**DOI:** 10.1371/journal.pone.0084109

**Published:** 2013-12-17

**Authors:** Tora Sund Morken, Axel Karl Gottfrid Nyman, Ioanna Sandvig, Sverre Helge Torp, Jon Skranes, Pål Erik Goa, Ann-Mari Brubakk, Marius Widerøe

**Affiliations:** 1 Department of Laboratory Medicine, Children’s and Women’s Health, Norwegian University of Science and Technology (NTNU), Trondheim, Norway; 2 Department of Circulation and Medical Imaging, Norwegian University of Science and Technology, Trondheim, Norway; 3 Department of Pediatrics, St. Olav University Hospital, Central Norway Regional Health Authority, Trondheim, Norway; 4 Department of Radiology, St. Olav University Hospital, Central Norway Regional Health Authority, Trondheim, Norway; Robert Debre Hospital, France

## Abstract

**Background:**

Neonatal intermittent hyperoxia-hypoxia (IHH) is involved in the pathogenesis of retinopathy of prematurity. Whether similar oxygen fluctuations will create pathological changes in the grey and white matter of the brain is unknown.

**Methods:**

From birth until postnatal day 14 (P14), two litters (total n = 22) were reared in IHH: hyperoxia (50% O_2_) interrupted by three consecutive two-minute episodes of hypoxia (12% O_2_) every sixth hour. Controls (n = 8) were reared in room-air (20.9% O_2_). Longitudinal MRI (Diffusion Tensor Imaging and T_2_-mapping) was performed on P14 and P28 and retinal and brain tissue were examined for histopathological changes. Long-term neurodevelopment was assessed on P20 and P27.

**Results:**

Mean, radial and axial diffusivity were higher in white matter of IHH versus controls at P14 (*p* < 0.04), while fractional anisotropy (FA) was lower in the hippocampal fimbria and tended to be lower in corpus callosum (*p* = 0.08) and external capsule (*p* = 0.05). White matter diffusivity in IHH was similar to controls at P28. Higher cortical vessel density (*p* = 0.005) was observed at P14. Cortical and thalamic T_2_-relaxation time and mean diffusivity were higher in the IHH group at P14 (*p* ≤ 0.03), and albumin leakage was present at P28. Rats in the IHH group ran for a longer time on a Rotarod than the control group (*p* ≤ 0.005). Pups with lower bodyweight had more severe MRI alterations and albumin leakage.

**Conclusion:**

IHH led to subtle reversible changes in brain white matter diffusivity, grey matter water content and vascular density. However, alterations in blood-brain barrier permeability may point to long-term effects. The changes seen after IHH exposure were more severe in animals with lower bodyweight and future studies should aim at exploring possible interactions between IHH and growth restriction.

## Introduction

Preterm born infants may be subject to pathological conditions in several organ systems like retinopathy of prematurity (ROP) and preterm encephalopathy. The severity of ROP is a predictor of neurodevelopmental outcome [1] and parallels postnatal head growth deficit [2]. The retina has been called a “window to the brain” since it is an extension of brain tissue [3], and it is conceivable that environmental insults during critical periods in development may contribute to both ROP and preterm encephalopathy. Exposure to unphysiological levels of oxygen may be such an environmental insult. In the retina it is the *fluctuations* and *range* between high (hyperoxic) and low (hypoxic) levels of oxygen that create ROP rather than either exposure alone [4,5], and even small fluctuations in oxygen concentration around a normoxic mean induce pathological changes [6]. Hyperoxia due to treatment combined with hypoxia due to apnoea is common in the sick premature child, and clinicians question whether such unphysiological oxygen levels will also affect long-term brain development [7]. In animal models this has been explored after neonatal hypoxia [8-10] or hyperoxia [11,12]. However, brain development following neonatal fluctuating oxygen levels, an exposure that would more closely mimic a clinical setting, is largely unexplored. 

In ROP the coordinated formation of the neurovascular unit comprising neurons, endothelial cells, pericytes and astrocytes is disrupted [13]. Obliteration of immature vessels by hyperoxia leads to tissue hypoxia that in turn stimulates uncontrolled neovascularization, microbleedings, and eventually injury to the rapidly growing neural tissue of the retina [14]. The neurovascular unit may also be a target in the brain of preterm born children, a population where long-term pathological changes in grey and white matter are abundant [15,16]. Periventricular white matter is poorly vascularized due to arterial end-zones, and disturbances of vascular supply is likely involved in the development of periventricular leukomalacia (PVL) [17]. Furthermore, paucity of endothelial pericyte [18] and astrocyte coverage [19] in the germinal matrix of preterm born infants, a predilection site for intraventricular haemorrhage, indicates fragile vasculature. Vascularization of both the retina [20] and brain [21] occur in parallel to the brain growth spurt from gestational week 20 until term in humans and from birth until postnatal day 14 (P14) in the rat, a species that at birth is comparable to a very preterm human infant [22]. Both intermittent and continuous neonatal hypoxia reduce long-term myelination and induce angiogenesis in the rat brain [10,23]. Furthermore, neonatal hyperoxia causes microvascular degeneration in grey matter [24] and maturation-dependent cell death in white matter [25,26]. It is conceivable that, both in the retina and brain, fluctuations between high and low oxygen levels disturb the coordinated formation of the neurovascular unit in a period of major growth and differentiation in both organs. Therefore, exposure of neonatal rats to a profile of intermittent hyperoxia-hypoxia (IHH) that cause ROP may induce a parallel disruption of neural and vascular tissue in the brain with ensuing white and grey matter injury. 

To investigate this hypothesis we conducted a study of long-term brain development in rats exposed to IHH from birth until P14. Outcome was evaluated with multimodal *in vivo* magnetic resonance imaging (MRI) combined with neurodevelopmental testing and detailed immunohistochemical studies of blood-brain barrier (BBB) permeability and vascular density.

## Methods

### Ethics Statement

Experiments were conducted in accordance with Guidelines set by the Norwegian Ethics Committee for Animal Research and approved by the responsible governmental authority (Forsøksdyrutvalget, permit no: 3748). 

### Oxygen Profiling

**Figure 1:**
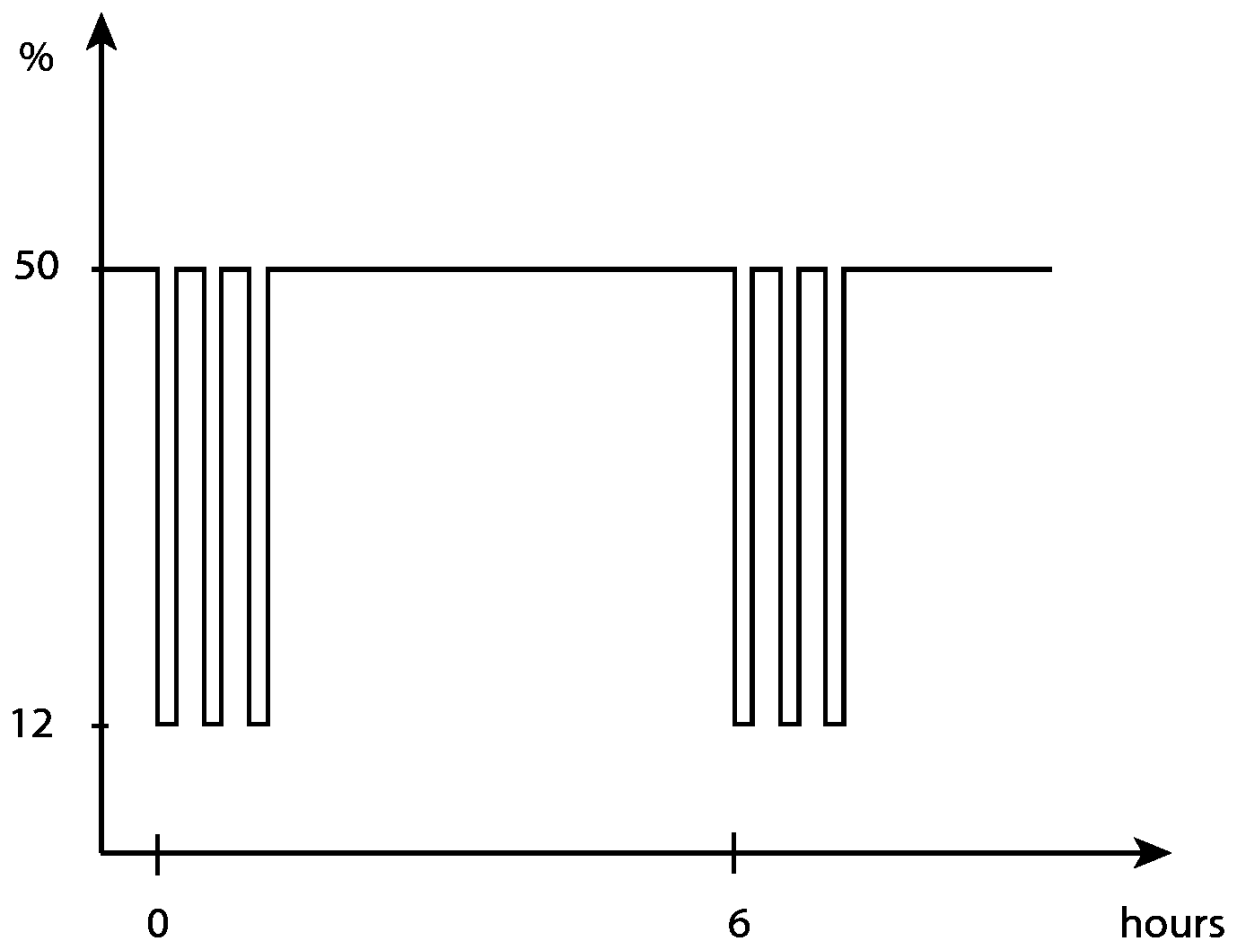
Experimental profile of intermittent hyperoxia-hypoxia. Every sixth hour of hyperoxia (50% O_2_) was interrupted by a cluster of three consecutive episodes of hypoxia (12% O_2_) ten minutes apart, each of two minutes. This profile was applied continuously from within four hours after birth until postnatal day 14.

An A84 Oxycycler (Biospherix Ltd., Lacona, NY) was used to program a profile of IHH that create ROP in neonatal rats [27]. Continuous hyperoxia (50% O_2_) was interrupted every sixth hour by three consecutive two-minute long episodes of hypoxia (12% O_2_), each ten minutes apart (Figure 1). The ramp time between hyperoxia and hypoxia was approximately two minutes. Bedding was changed during a 50% cycle on day seven. The chamber was then opened for less than three minutes. Inside the chamber, oxygen was controlled via a sensor and a computerized feed-back system where nitrogen was used to lower and oxygen was used to raise the oxygen concentration. CO_2_, temperature and relative humidity was continuously monitored and kept within physiological levels via the use of Soda lime (Anmedic AB, Stockholm, Sweden), ventilation and a built-in fan. 

### Experiment Groups

Time-mated Sprague-Dawley rats were purchased from Scanbur AS (Nittedal, Norway). Within four hours after delivery of the last pup, litters with dam were placed in specialized controlled oxygen chambers in their cages (A-30524, Biospherix Ltd.). To ensure a uniform age at start of exposure whole litters were exposed from birth. Two litters (A: n = 13 and B: n = 9) were exposed to IHH while one litter (controls: n = 8) was exposed to room-air (20.9% O_2_) from P0 to P14. Animals were kept on a 12:12 hours light: dark cycle and had food and water *ad libitum*. Half the animals were euthanized on P14 and the other half on P28 after MRI and neurodevelopmental testing. 

### Magnetic Resonance Imaging

MRI was performed on P14 (IHH: n = 22; control: n = 8) and P28 (IHH: n = 12; control: n = 4) using a 7T magnet (Biospec 70/20 AS, Bruker Biospin MRI, Ettlingen, Germany) with water‑cooled (BGA-12, 400 mT/m) gradients. All animals were imaged longitudinally until euthanization. A volume resonator was used for radiofrequency (RF) transmission and actively decoupled head surface coils were used for RF reception. During scanning the anaesthetized (2% isoflurane in 30% O_2_/70% N_2_) pups lay prone in a dedicated water-heated bed (Bruker Biospin MRI) and the head of every animal was fixated in the same position with tooth-bar, nose-mask and polystyrene. Temperature and respiration were monitored during the scanning procedure. On P14 and P28 coronal T_2_-maps were obtained with a turbo spin‑echo (RARE) sequence: Effective TE = 16, 48, 80, 120 ms; TR = 3750 ms; RARE-factor = 4; FOV = 25 x 20 mm^2^; MTX = 160 x 96 reconstructed to 164 x 128; 15 slices á 0.75 mm. Diffusion tensor imaging (DTI) was performed with an EPI sequence using 30 directions and b = 1000 ms; 5 b0 images and TE = 32; TR = 3750 ms; FOV = 25.6 x 25.6 mm^2^; MTX = 128 x 128 and 15 coronal slices á 0.75 mm. 

### Magnetic Resonance Image Analyses

In-house developed software was used for calculating T_2_-maps (MATLAB ver. R2010a, Math Works Inc., Natick, MA), and ImageJ (ver. 1.42q, National Institute of Health, Bethesda, MD) was used for image analyses. T_2_-maps were calculated by fitting a mono-exponential model to the signal intensity of the images with different TE-values. Regions of interest (ROI) were drawn in the parasagittal cortex, thalamus, hippocampus, putamen and corpus callosum in two slices corresponding to –3.25 mm and –1.5 mm from the bregma [28] (Figure S1). For brain volume measurements the areas of both hemispheres were drawn manually on the T_2_-maps in each image slice and the total brain volumes were calculated and compared between groups. DTI-analyses were performed with the tools of the FMRIB software library (FSL ver. 4.1.4, Oxford Centre for Functional MRI of the Brain, Oxford, UK; www.fmrib.ox.ac.uk/fsl). Images were pre-processed to reduce artefacts due to motion and eddy current distortions by affine transformation and co-registration of the diffusion encoded images to the b0 images. Brains were segmented out using the Brain Extraction Tool before FDT ver. 2.0 (FMRIB’s Diffusion Toolbox; both part of FSL) was used to fit a voxelwise diffusion tensor model to the diffusion image data. Maps for the fractional anisotropy (FA), mean, radial and axial diffusivity were created. To limit partial volume effects, ROI were manually drawn in the centre of white matter structures at all relevant image slices on the FA maps and combined to five volumes of interest: corpus callosum (body), external capsule, internal capsule, hippocampal fimbriae and white matter (comprising all of the aforementioned areas, see Figure S1). Mean FA, radial, axial and mean diffusivity were calculated in each of these volumes of interest in each animal. Due to low signal-to-noise-ratio (SNR) in the deeper brain structures in the diffusion‑weighted images on P28, masks were only drawn in the body of the corpus callosum where SNR was adequate for robust calculations of DTI-metrics at this time-point (IHH: n = 6, control: n = 4).

### Rotarod

On P20 and P27, rats were tested on a Rotarod (Bioseb, Vitrolles, France) in four consecutive trials with increasing rotation from 0 to 40 rpm within two minutes. The time the animals ran on the rotating rod was recorded. 

### Histology

Rats were euthanized by an overdose of pentobarbital (300 mg/kg; Vétoquinol, Lure Cedex, France). For fixation an intracardial perfusion of 4% paraformaldehyde (Fluka Chemie AG, Buchs, Switzerland) in phosphate-buffered saline (Oxoid Limited, Hampshire, UK) was given. Eyes were enucleated and the orbita were inspected for gross abnormalities. Thereafter the retina of the left eye was dissected and whole-mounted onto Super Frost object glass (Thermo Fisher Scientific Inc., Waltham, MA) while the right eye and the brain were paraffin-embedded. Only a few retinas were successfully whole-mounted in each group (IHH: n = 2; control: n = 1). Coronal brain sections corresponding to –3.25 mm from the bregma [28] and retinal cross-sections (4 μm) were cut and stained with Haematoxylin & Eosin (H&E; Cell Path Ltd., Newtown, UK and Sigma-Aldrich Inc., St. Louis, MO) and examined for morphological changes.

### Immunohistochemistry

Brain sections were incubated with anti-rat-albumin (1:16000) (Nordic Immunology, Eindhoven, the Netherlands) for albumin leakage, Biotinylated *Griffonia* (*Bandeiraea*) *Simplicifolia* Lectin I Isolectin B4 (1 μg/ml) (Vector Laboratories Inc., Burlingame, CA) for vascular density, anti-glial fibrillary acid protein (GFAP; 1:200) (Cymbus Biotechnology, Southampton, UK) for astrogliosis and anti-ED1-FITC (1:400) (Serotec, Raleigh, NC) for cluster of differentiation 68 (CD68) positive activated microglia. Sections were then incubated with labelled polymer HRP anti-rabbit (Dako A/S, Glostrup, Denmark), streptavidin-conjugated FITC (Vector Laboratories Inc.), horse-anti-mouse-biotin (Vector Laboratories Inc.) and rat-anti-FITC-biotin (Roche, Basel, Switzerland). Visualization was performed using a Vectastain ABC kit (Vector Laboratories Inc.) and a Diaminobenzidine (DAB) kit (Vector Laboratories Inc.). Images of brain sections were captured with a MIRAX MIDI system (Carl Zeiss MicroImaging GmbH, Jena, Germany) and examined. The presence (score = 1) or not (score = 0) of increased albumin immunoreactivity in the neuropil in the hippocampus, cortex, thalamus and hypothalamus was scored. Vessel density was quantified in three areas of the cortex (parietal and temporal), thalamus, hippocampus (CA2, CA3 and dentate gyrus) and periventricular white matter at x400 magnification using ImageJ and normalized relative to control. The result was averaged for each animal and the mean was compared between groups. GFAP and CD68-stained sections were evaluated qualitatively for changes between groups. Following incubation with Isolectin B4 (12.5 μg/ml) overnight and thereafter with streptavidin-conjugated FITC, retinal wholemounts were analyzed qualitatively for neovascularization and maturation of the retinal vascular bed. 

### Statistical Analysis

To test for differences in mean between experiment groups and between time-points in the same individual, paired and unpaired two-tailed Student’s *t*-tests were applied, respectively. Between-litter differences were analyzed with one-way ANOVA followed by post-hoc tests with Bonferroni correction for multiple comparisons. A *p* < 0.05 was chosen as level of significance. Data are presented as mean ± 95% confidence interval. Analyses were performed in SPSS ver. 19.0 (SPSS Inc., Chicago, IL).

## Results

### Retinal Changes

**Figure 2:**
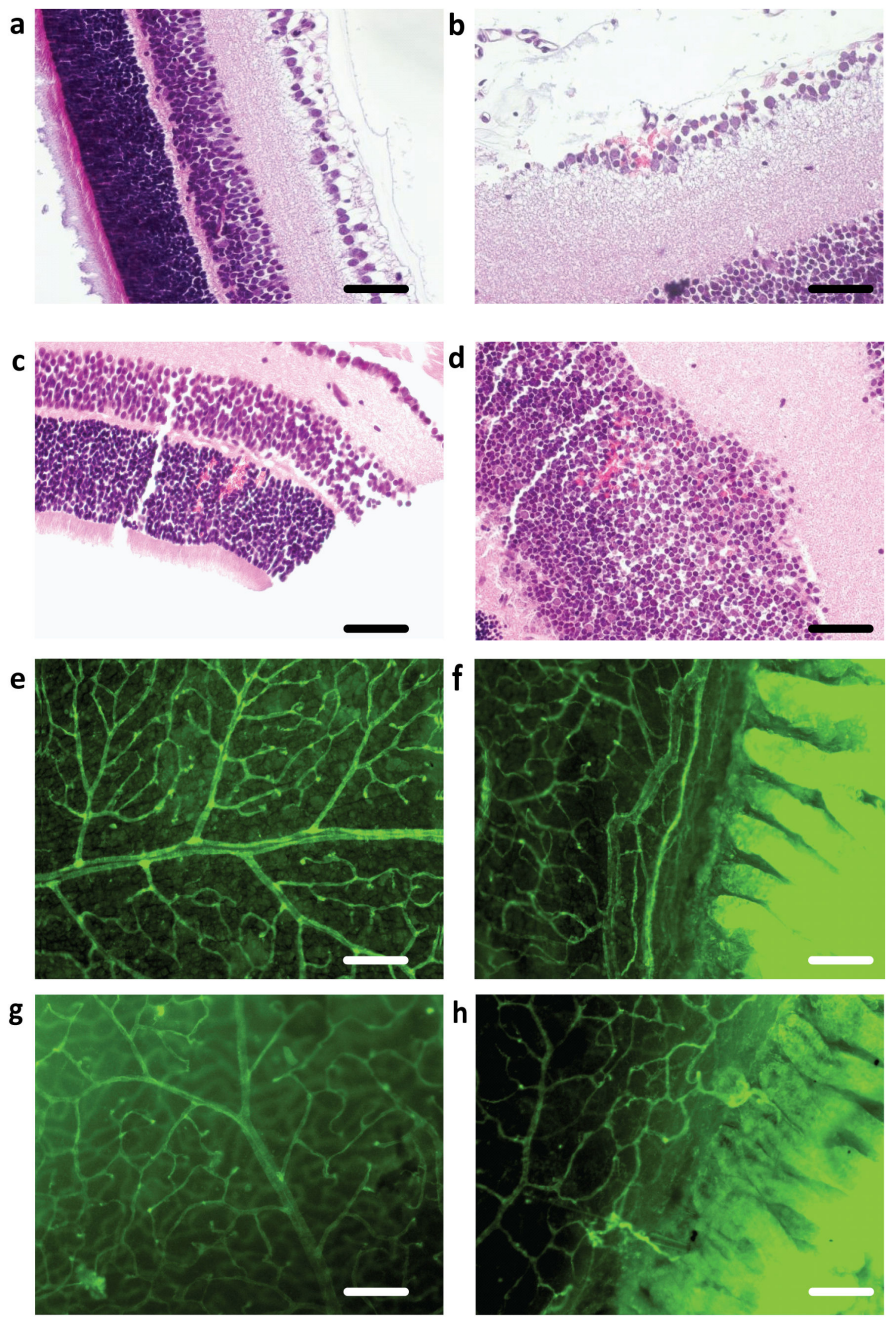
Retina. (a-d): H&E retinal slices from control (a) and IHH animals at P14 (b-d) showing haemorrhage in the ganglion cell layer (b), inner nuclear layer (c) and outer nuclear cell layer (d). (e-h): Retinal wholemounts stained with endothelial-specific Biotinylated *Griffonia* (*Bandeiraea*) *Simplicifolia* Lectin I Isolectin B4 from controls at P28 with a mature vascular bed (e) and no vasculature extending beyond the ora serrata (f). Retinal vascular bed in IHH animal at P28 with less remodelling (g) and areas of vascularization beyond the ora serrata (h). (a-d): x400 magnification; scale bar = 50 μm. (e-h): x100 magnification; scale bar = 200 μm. Abbreviations, IHH, intermittent hyperoxia-hypoxia; P14, postnatal day 14; P28, postnatal day 28.

At P14 orbital macroscopic bleedings were observed in four out of ten IHH animals and in none of the controls. Furthermore, haemorrhage in the ganglion cell layer and/or inner and outer nuclear layer were seen in five out of nine animals in the IHH group, often in all three layers and on several locations (Figure 2 a-d). Similar bleedings were observed in one control animal in one location. At P28 areas of neovascularization were observed in retinal wholemounts of both IHH animals (Figure 2 g-h) but not in the control (Figure 2 e-f), and the control retinal vasculature appeared more remodelled compared to IHH. 

### Bodyweight and Brain Volume

On P14 litter A weighed less (n = 13, mean = 21.2 ± 0.6 g) than litter B (n = 9, mean = 27.4 ± 0.5 g) and controls (n = 8, mean = 25.8 ± 1.1 g; *p* < 0.01) while B was heavier than controls (*p* = 0.02). At P28 mean weight of B (79.6 ± 2.8 g) was higher than that of A (73.9 ± 3.0 g, *p* = 0.02), but none were different from mean weight of controls (76.3 ± 3.4 g). On P14, litter A (814.5 ± 20.7 μl) had lower brain volume than litters B (871.8 ± 28.9 μl) and C (875.4 ± 17.9 μl; *p* < 0.01) while litter B and C did not differ. At P28 mean brain volume of A (1002.0 ± 33.5 μl,) was lower than that of B (1081.2 ± 8.1 μl, *p* < 0.01), but none were different from controls (1049.4 ± 29.6 μl, *p* ≥ 0.05). The ratio of brain volume over bodyweight in litter A at P14 was 38.4 ± 1.3, which was higher than in litter B (31.8 ± 1.1) and litter C (34.1 ± 2.0, *p* < 0.01). At P28 this ratio was similar between litters: 13.6 ± 0.7 in litter A, 13.6 ± 0.7 in litter B and 13.8 ± 0.9 in litter C.

### Histopathology

No pathological changes were seen in the H&E brain sections with regards to neuropil, neurons, glial cells, vessels or leptomeninges. There were especially no extravasations of erythrocytes, inflammation or eosinophilic degeneration in neurons observed. 

### Immunohistochemistry

**Figure 3:**
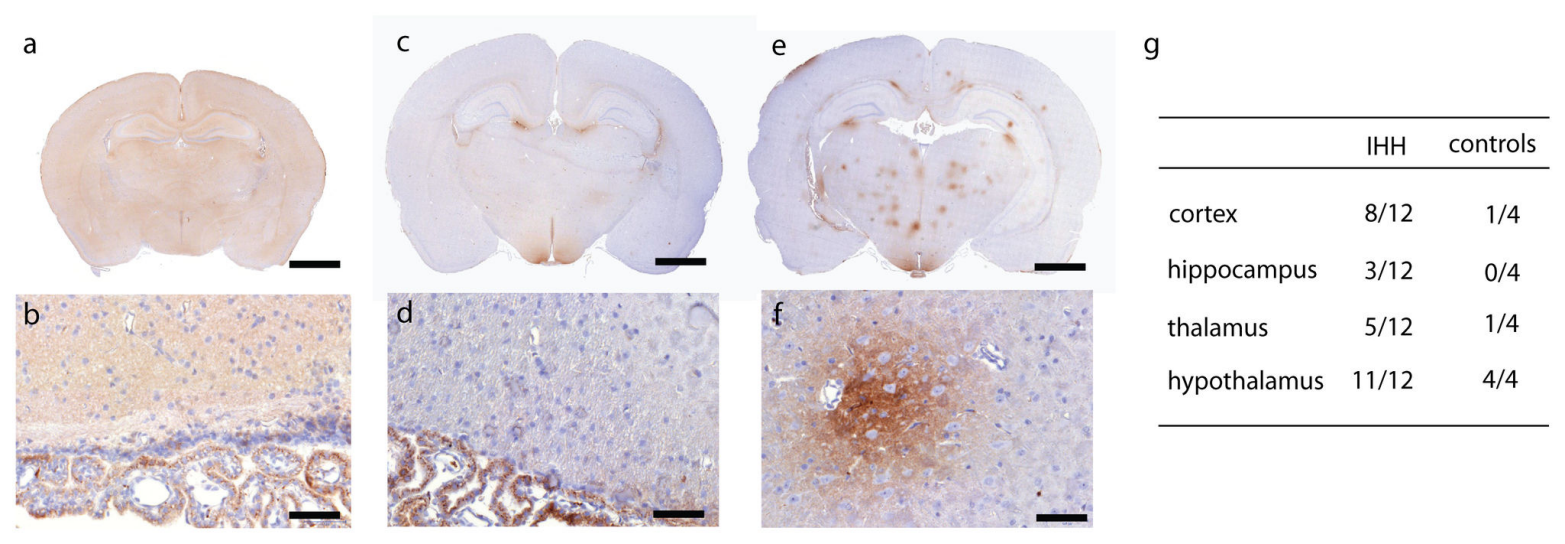
Albumin. Albumin immunoreactivity (brown stain) at P14 (a-b) and P28 in controls (c-d) and IHH (e-f). Note positive immunoreactivity for albumin in both experiment groups at P14 and several spots of albumin immunoreactivity in the cortex and thalamus of the IHH animal at P28. Albumin is present in intracellular vesicles in neuroependymal cells in the ventricles (b & d). (g) The sum of positive scores for albumin leakage in the neuropil of the respective brain areas are presented as fractions of the maximum possible score in each experiment group at P28. (a, c & e): scale bar = 2 mm (b, d & f): scale bar = 50 μm at x400 magnification. Abbreviations: IHH, intermittent hyperoxia-hypoxia; P14, postnatal day 14; P28, postnatal day 28.

**Figure 4:**
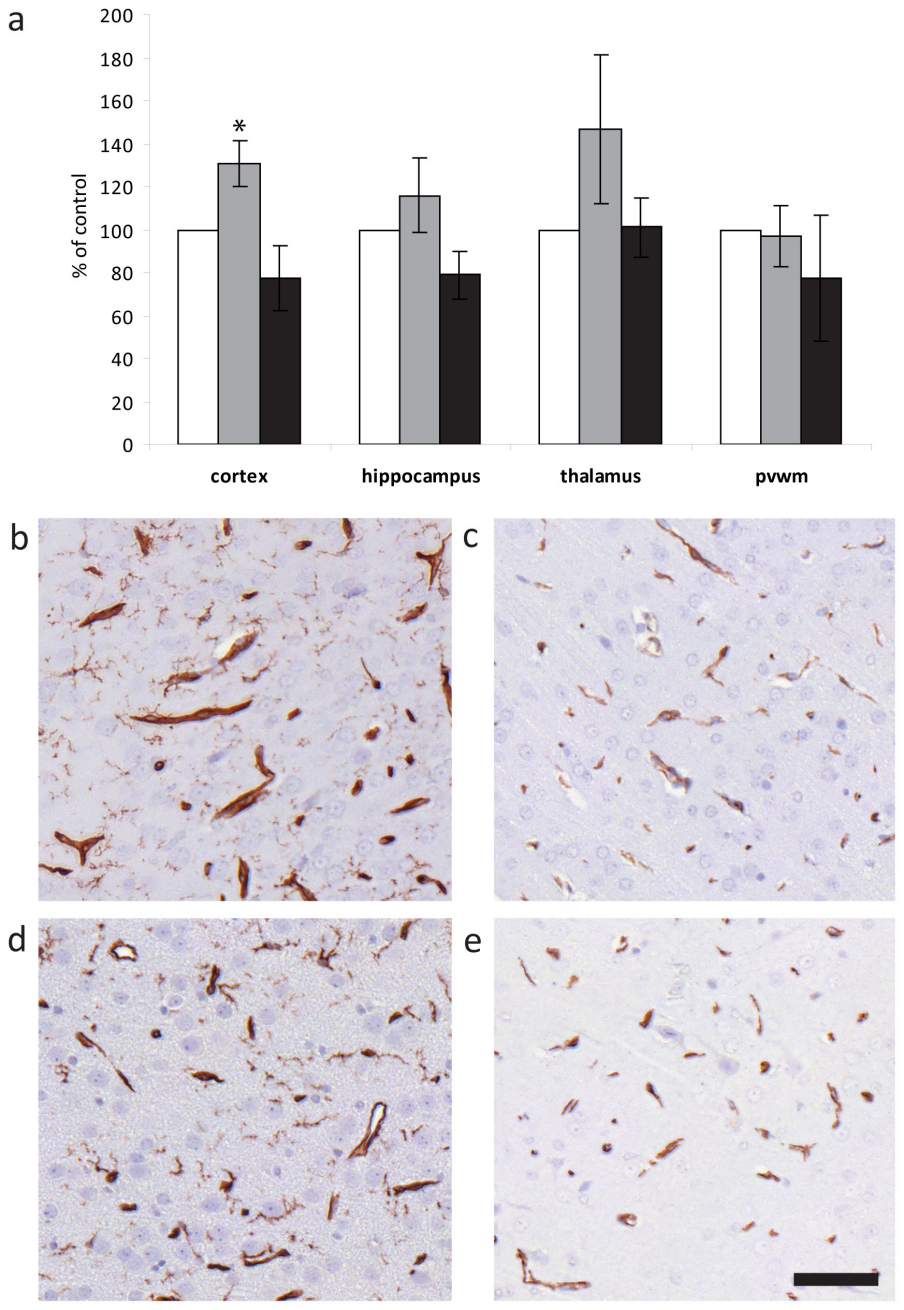
Vascular density. (a) Vascular density as % of controls (white columns, n = 4) in IHH animals at P14 (grey columns; IHH: n = 10) and P28 (black columns; IHH: n = 12). Data are expressed as mean ± 95% confidence intervals. (b-e) x400 magnification of lectin-stained endothelium in IHH (upper row) and control (lower row) at P14 (b & d) and P28 (c & e). *p = 0.005 IHH vs. control. Scale bar = 50 μm. Abbreviations: IHH, intermittent hyperoxia-hypoxia; P14, postnatal day 14; P28, postnatal day 28; pvwm, periventricular white matter.

A generalized positive immunoreaction for albumin was seen in both IHH and controls in the neuropil at P14 and in the hypothalamus, a circumventricular organ void of BBB [29], at P28 (Figure 3 a & c). However, in the IHH group focal perivascular albumin leakage above control levels was observed in the neuropil at P28 (Figure 3 e & f). Although albumin leakage was present in most animals in both litters in the IHH group (A: 6/7 animals versus B: 3/5 animals), litter A had multiple larger areas (Figure 3 e), while those of litter B were singular smaller areas similar to those observed in control animals. Cortical vascular density of the IHH animals was higher than controls at P14 (*p* = 0.005, see Figure 4) and tended to be higher in the thalamus (*p* = 0.07). At P28 vascular density was similar to controls. GFAP staining was not elevated in the IHH versus controls, nor was there increased activation of microglia at neither time-point (data not shown). 

### T_2_ Maps

**Figure 5:**
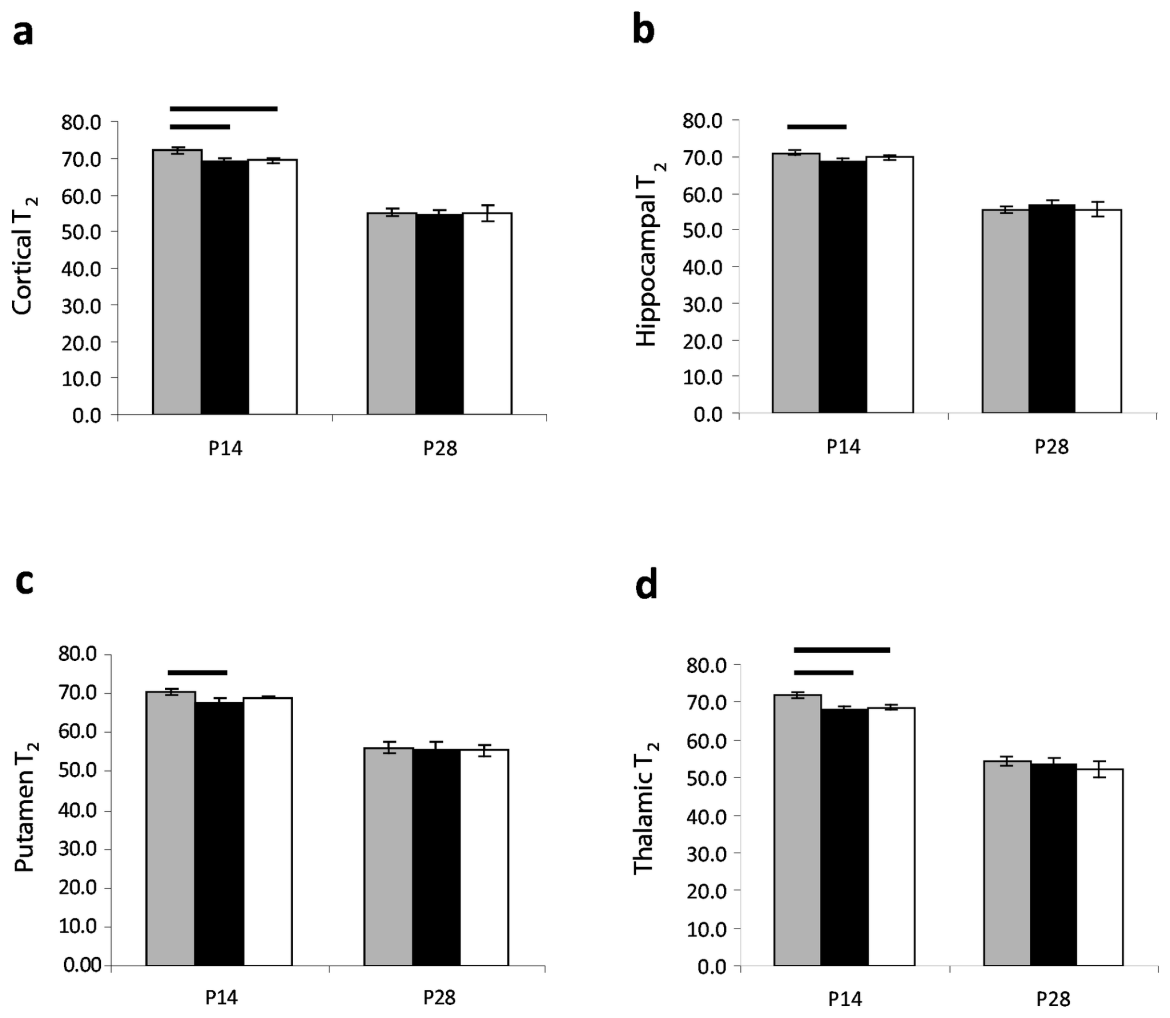
Litter differences in T_2_. T_2_-relaxation time in the brain at P14 in litter A (grey columns, n = 13), litter B (black columns, n = 9) and C (white columns, n = 8) in the cortex (a), hippocampus (b), putamen (c) and thalamus (d). T_2_-relaxation times are shown in milliseconds. Differences between litters are marked where significant (*p* < 0.05). Data are presented as mean ± 95% confidence interval. Abbreviations: IHH, intermittent hyperoxia-hypoxia; P14: postnatal day 14; P28: postnatal day 28.

Visual evaluation of T_2_-maps did not reveal differences in grey or white matter. However, at P14 the measured T_2_-relaxation time was higher in the IHH group than in controls in cortex (*p* = 0.01) and thalamus (*p* = 0.03) (Table 1), but similar on P28. In both groups T_2_-relaxation time decreased from P14 to P28 (*p* < 0.003). At P14 litter A had a higher mean T_2_ relaxation time than litter B in all areas (*p* < 0.006) and than controls in cortex and thalamus (*p* < 0.001, Figure 5). There were no differences in T_2_-relaxation time in corpus callosum at P14 between experiment groups (IHH: 78.4 ± 0.7 vs. controls: 78.3 ± 4.0, *p* = 0.7) and P28 (IHH: 55.6 ± 1.3 vs. controls: 54.6 ± 3.6, *p* = 0.5). 

**Table 1:** T_2_ relaxation time and mean diffusivity.

		**cortex**		**hippocampus**		**putamen**		**thalamus**	
**Mean diffusivity (mm^2^/s)**									
**P14**	**IHH**	0.00105	± 0.00003*	0.00104	± 0.00003	0.00104	± 0.00003	0.00104	± 0.00003*
	**litter A**	0.00106	± 0.00004*	0.00107	± 0.00003*	0.00108	± 0.00003	0.00106	± 0.00003*§
	**litter B**	0.00102	± 0.00009	0.00100	± 0.00009	0.00099	± 0.00007	0.00101	± 0.00009
	**Control**	0.00098	± 0.00005	0.00098	± 0.00005	0.00100	± 0.00004	0.00098	± 0.00005
**T_2_-relaxation time (msec)**									
**P14**	**IHH**	70.7	± 0.8*	69.9	± 0.9	69.3	± 0.9	70.2	± 1.0*
	**Control**	69.3	± 0.7	69.7	± 1.1	69.0	± 0.2	68.5	± 1.1
**P28**	**IHH**	54.9	± 0.8 †	56.0	± 0.6 †	55.9	± 1.3 †	54.1	± 0.8 †
	**Control**	54.9	± 2.1†	55.6	± 2.4 †	55.4	± 1.4 †	52.2	± 1.2 †

T_2_-relaxation time and mean diffusivity in grey matter at P14 (IHH: n = 22; control: n = 8) and P28 (T_2_-relaxation time; IHH: n = 12; control: n= 4). Data are presented as mean ± 95% confidence interval. *p < 0.05 vs. control. § *p* < 0.05 litter A vs. litter B. †*p* < 0.003 P14 vs. P28 within each experiment group. Abbreviations: IHH, intermittent hyperoxia-hypoxia; P14: postnatal day 14; P28: postnatal day 28.

### Diffusion Tensor Imaging

**Figure 6:**
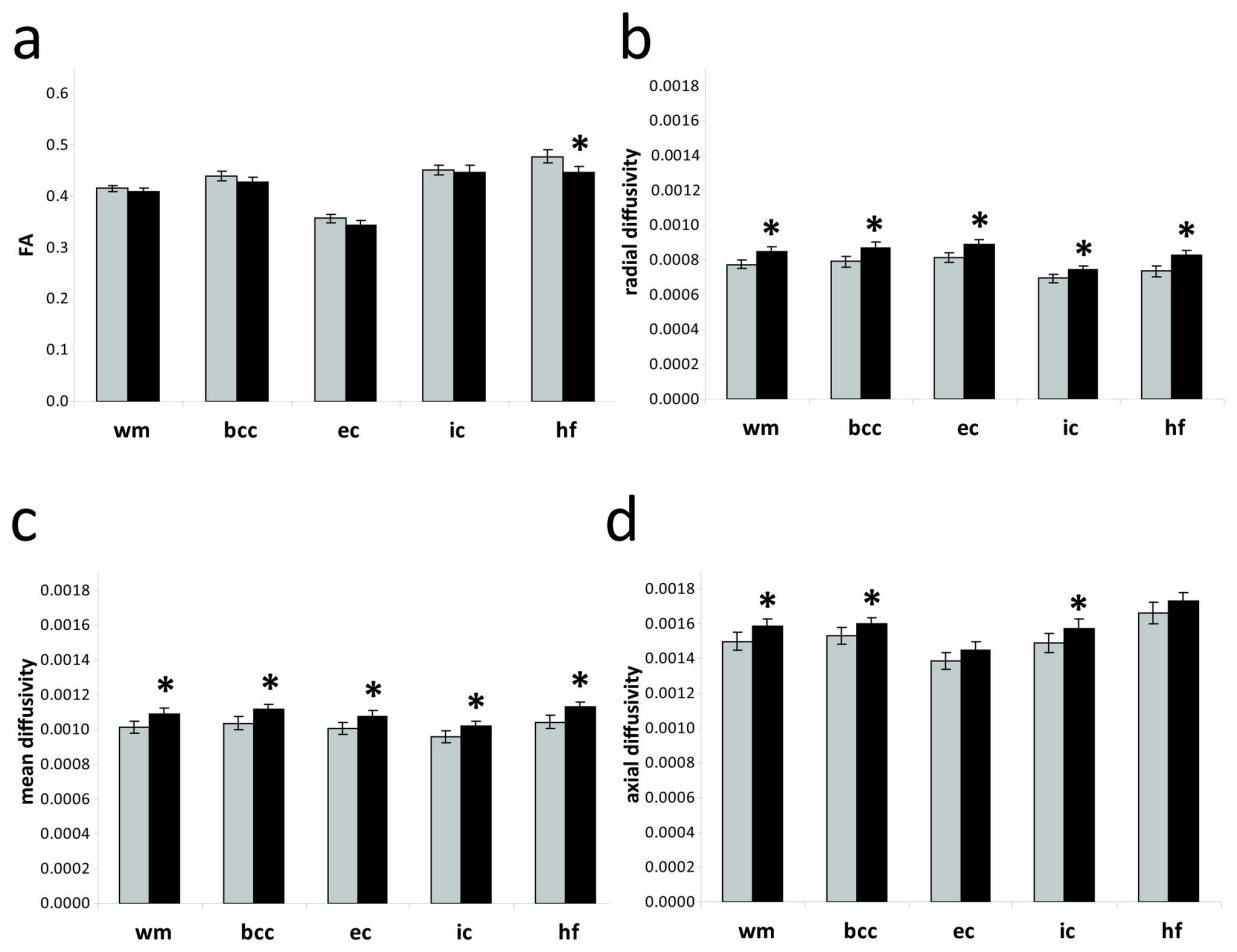
DTI on postnatal day 14. Fractional anisotropy (a); radial (b); mean (c) and axial diffusivity (d) in white matter areas of controls (grey columns, n = 8) and IHH (black columns, n = 22). Mean, axial and radial diffusivity are shown in units of mm^2^/s. Data are presented as mean ± 95% confidence interval.* *p* < 0.04. Abbreviations: wm: all of white matter; bcc: corpus callosum (body); ec: external capsule; ic: internal capsule; IHH, intermittent hyperoxia-hypoxia; hf: hippocampal fimbriae.

**Figure 7:**
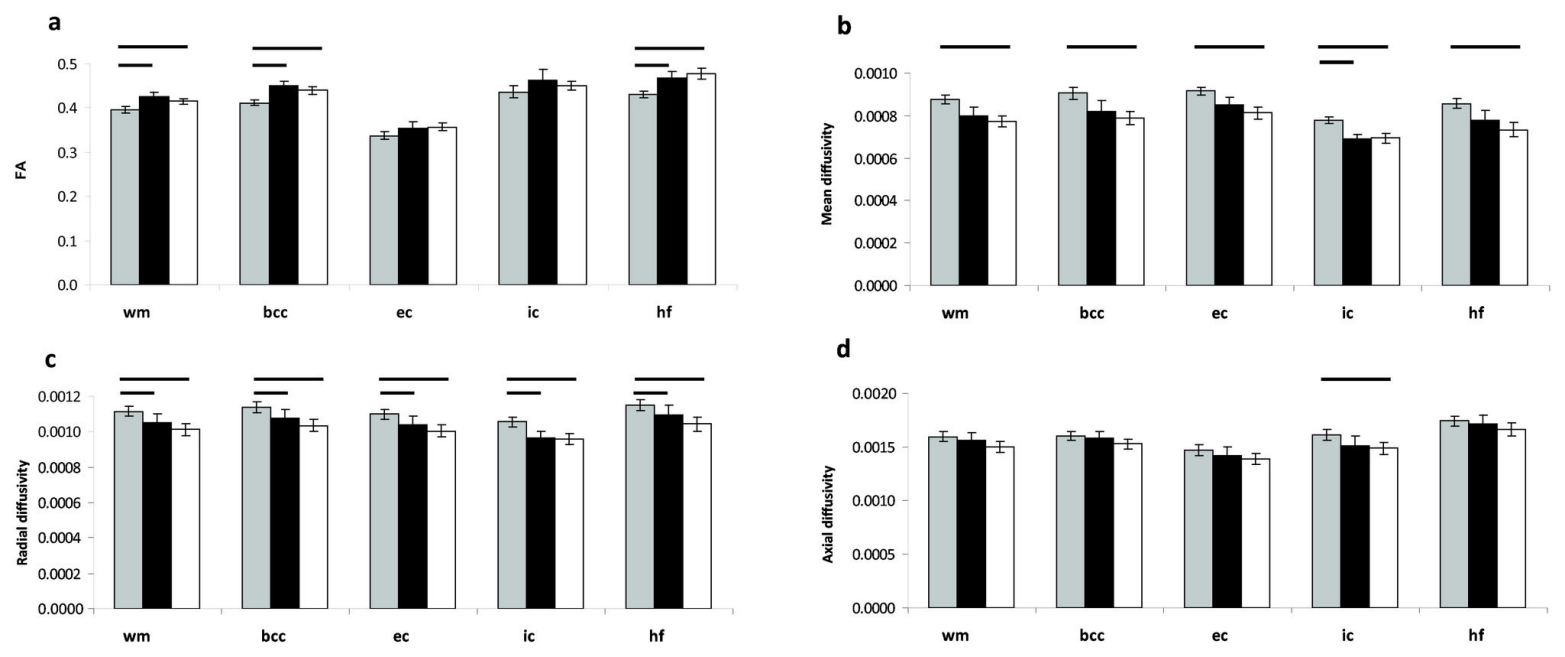
Litter differences in DTI metrics. Fractional anisotropy (a); mean (b), radial (c) and axial diffusivity (d) in white matter areas of litter A (grey columns, n = 13), B (black columns, n = 9) and controls (white columns, n = 8) on postnatal day 14. Mean, axial and radial diffusivity are shown in units of mm^2^/s. Data are presented as mean ± 95% confidence interval. Differences between litters are marked where significant (*p* < 0.05). Abbreviations: wm: all of white matter; bcc: corpus callosum (body); ec: external capsule; ic: internal capsule; IHH, intermittent hyperoxia-hypoxia; hf: hippocampal fimbriae.

At P14, mean, radial and axial diffusivity were higher in the IHH than in the control group in all areas of white matter (*p* < 0.04) except axial diffusivity in the external capsule (*p* = 0.09) and in hippocampal fimbriae (*p* = 0.08). Fractional anisotropy (FA) was lower in the hippocampal fimbriae (*p* = 0.001) and tended to be lower in the external capsule (*p* = 0.05) and corpus callosum (*p* = 0.08, see Figure 6) in the IHH group. At P28 there was no difference in white matter diffusivity of the corpus callosum between IHH and controls, but there was a significant increase in FA (*p* = 0.03) and decrease in radial (*p* = 0.008) and mean (*p* = 0.009) diffusivity from P14 to P28 in the IHH group (Table 2). Litter A had a lower mean FA in all of white matter, corpus callosum and hippocampal fimbria than B and controls (*p* < 0.02, Figure 7). Furthermore, radial diffusivity in A was higher than that of B and controls in all areas (*p* < 0.01). Mean diffusivity for litter A was higher than litter B in the internal capsule (*p* = 0.001) and in all areas compared to controls (*p* < 0.005). Axial diffusivity in the internal capsule was higher in litter A than controls (*p* = 0.04). At P28 there were no differences in DTI parameters between litters (data not shown). Grey matter mean diffusivity in cortex and thalamus were higher at P14 in IHH than in controls (Table 1). Litter A had a higher mean diffusivity than controls in all white matter areas and than litter B in thalamus, while litter B was not significantly different from controls in any area. At P28 there were no differences in mean diffusivity in cortex between groups (IHH: 0.00101 ± 0.00003 mm^2^/s vs. controls: 0.00098 ± 0.00002 mm^2^/s, *p* = 0.1) but litter A (0.00104 ± 0.00005 mm^2^/s) had a higher mean diffusivity than controls (*p* = 0.04). Litter B (0.00099 ± 0.00004 mm^2^/s) was not significantly different from either litter A or controls.

**Table 2:** Diffusion tensor metrics of the corpus callosum at postnatal day 14 and 28.

		FA		radial diffusivity		mean diffusivity		axial diffusivity	
				mm^2^/s					
P14	IHH	0.44	± 0.02	0.00090	± 0.00005*	0.00115	± 0.00005*	0.00166	± 0.00004*
	Control	0.44	± 0.02	0.00078	± 0.00002	0.00103	± 0.00001	0.00153	± 0.00003
P28	IHH	0.52	± 0.05†	0.00072	± 0.00005†	0.00103	± 0.00003†	0.00164	± 0.00005
	Control	0.48	± 0.05	0.00077	± 0.00005	0.00103	± 0.00004	0.00154	± 0.00009

DTI metrics in animals with DTI of the corpus callosum at both P14 and P28 in IHH (n = 6) and controls (n = 4). Data are presented as mean ± 95% confidence interval. *p < 0.02 IHH vs. control, †*p* ≤ 0.03 P14 vs. P28. Abbreviations: FA, fractional anisotropy; IHH, intermittent hyperoxia-hypoxia; P14, postnatal day 14; P28, postnatal day 28.

### RotaRod

**Figure 8:**
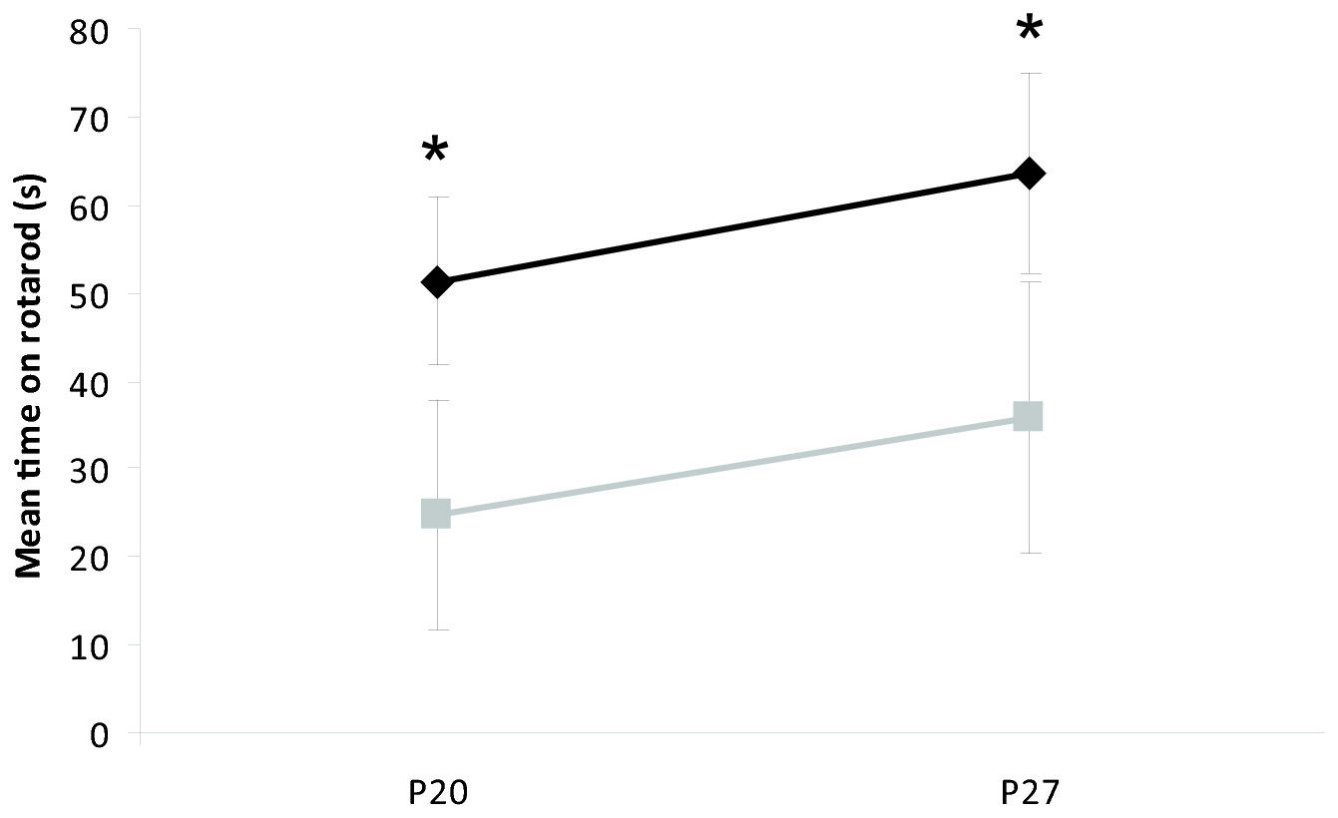
Rotarod testing. Mean time on a Rotarod on P20 and P27 of IHH (n = 12, black diamonds) and controls (n = 4, grey boxes). *p ≤ 0.03 IHH vs. control. Data are presented as mean ± 95% confidence intervals. Abbreviations: IHH, intermittent hyperoxia-hypoxia; P20, postnatal day 20; P27, postnatal day 27.

The IHH group ran for a longer time on the RotaRod than controls at both P20 (*p* = 0.003) and P27 (*p* = 0.005, Figure 8). 

## Discussion

### White Matter

During the first postnatal weeks the white matter of rats undergoes maturational changes, such as increase in axon calibre, axon packing, oligodendrocyte coverage and myelination, which affects the free water diffusion mainly by hindering water diffusion perpendicular to the white matter axons [30]. DTI is an excellent method to study directional free water diffusion and thereby *in vivo* white matter microstructural development and myelination [31]. In our study we used DTI to evaluate white matter and found higher mean, axial and radial diffusivity together with a lower FA among IHH animals than controls at P14. The high mean diffusivity can be explained by increased water diffusivity in all directions reflected in the concurrent increase in both axial and radial diffusivity. Such an overall increase in water diffusivity would occur in extracellular (vasogenic) edema. However, an increase in extracellular water content would also increase T_2_ in white matter, which we did not find. Therefore the higher mean diffusivity possibly reflects a less hindered water diffusion that may indicate a less mature white matter, since mean diffusivity is known to decrease with white matter maturation. This decrease in mean diffusivity during brain development is probably caused by increasing complexity of the white matter tissue structures and the reduction in brain water content that occur with increasing age (reviewed by Hüppi et al. [32]). Similarly, our finding of higher radial diffusivity and low FA in white matter structures of IHH animals may also indicate less mature white matter, since radial diffusivity decreases and FA increases with maturation, reflecting the oligodendrocyte coverage and myelination of axons that hinder diffusion perpendicular to the axonal direction. 

A normalization of white matter diffusivity was observed from P14 to P28 following IHH exposure, and a similar normalization has earlier been reported following neonatal hypoxia-ischemia [33]. Albeit DTI data were limited at P28, we speculate that alterations in diffusivity after neonatal IHH may be related to white matter maturational delay rather than to permanent injury. Such an interpretation is supported by studies reporting that neonatal intermittent hypoxia delay the maturation of oligodendrocytes rather than injure axons permanently [34] and that neonatal hyperoxic exposure causes a similar maturational delay [35]. Delayed white matter maturation with subsequent hypomyelination is also observed in preterm infants with diffuse white matter injury [36]. However, our DTI findings contrast reports of permanent white matter diffusivity alterations after neonatal hypoxia [37] and hyperoxia [12]. Our profile of IHH had lower hyperoxia (50% versus 80%) and shorter hypoxia (three two-minute episodes every sixth hour versus ten days continuous exposure) than the aforementioned studies. This less severe exposure may have caused transient white matter affection with a preserved ability to catch-up maturation rather than a permanent injury, which may explain the observed normalization of white matter diffusivity after IHH exposure over time.

### Grey Matter

Higher grey matter T_2_-relaxation time as well as increased mean diffusivity following IHH indicates increased grey matter water content, and alterations in angiogenesis and BBB‑formation may explain the increased vascular density and albumin leakage long-term. The angiogenic effect of hypoxia [38] has been extensively studied in the adult, but not the neonatal brain [39]. On the other hand, hyperoxia cause microvascular degeneration in the neonatal brain [24] possibly inducing tissue hypoxia. By exposing the neonatal brain to a combination of high and low oxygen levels as in IHH, such vasoobliteration by hyperoxia may have potentiated the angiogenic effect of the hypoxic episodes. Moreover, the normalization of vascular density in the IHH group at P28 may possibly be mediated via apoptosis of the newly-formed endothelium [23,40]. However, the concomitant long-term albumin leakage indicates that BBB-integrity was permanently altered even though vessel density returned to normal levels. 

The decrease in T_2_-relaxation time in normal brain tissue during maturation of the brain coincides with a reduction in free extracellular water as neurons mature [41,42]. Although the BBB is not fully developed at neonatal age [43], most of the functional barrier properties i.e. glucose and albumin transporters are [29]. Thus the relatively high brain water content and high concentration of albumin in the cerebrospinal fluid at this age [29] may explain the observed albumin-immunoreactivity in the neuropil at postnatal day 14. The suggested presence of a transcellular route for albumin transport [44] is supported by visible albumin in intracellular vesicles in the choroid plexus cells at both P14 and P28 (Figure 3 b & d). 

### Rotarod

The IHH animals ran longer on the Rotarod than controls, contrary to what would be expected if IHH exposure had given motor function deficits. It is unlikely that this was caused by an improved motor function after IHH exposure. However, during consecutive trials several animals in the control group were able to turn around and apparently willingly jump off the rotating rod, thereby resulting in shorter time spent on the rod. None of the IHH animals showed this behaviour. Neonatal hyperoxia impair memory in rats [11] and although behavioural tests were not performed in the present study, we speculate that differences in memory may explain the observed longer time spent on a Rotarod after IHH exposure. 

### Retina

It should be noted that classic avascular zones with subsequent prominent neovascularization such as those seen in models for ROP with 24 hours exposure of alternating hyper- and hypoxia [4] have not been observed after IHH [27], or after fluctuating oxygen levels around a normoxic mean [6]. Nevertheless, in line with earlier reports [27], the highly clinically relevant profile of neonatal IHH in our study created retinopathy seen as macro- and microscopic haemorrhages. 

### Implications for Further Research

The strength of our study is the longitudinal design with MRI, immunohistochemistry and neurodevelopmental testing, enabling the comparison of *in vivo* grey and white matter changes to detailed histopathological characterization and long-term outcome. However, the exploration of brain development after neonatal IHH has raised several questions:

(I) Does intrauterine and postnatal growth influence susceptibility to injury from IHH? Rat pups born in larger litters have a lower bodyweight at birth and in the whole suckling period [45], indicating that intrauterine growth determines postnatal body weight gain. Even though no intervention was applied to induce growth restriction in our study, pups from the most numerous litter with the lowest bodyweight and brain volume had the most severe alterations in many parameters of brain development. This finding could have been caused by confounding maturational differences due to age with IHH exposure. However, because of rigorous control of the time of birth, the age of the pups was comparable in the present study. Nevertheless, lower body weight may lead to later achievement of developmental milestones [45], even though the higher brain volume/bodyweight ratio in the litter with the lowest bodyweight at P14 indicated that brain volume was relatively spared from a possible growth restriction. Such a maturational delay may have contributed to the differences observed in white matter diffusivity and in T_2_-relaxation time since, as mentioned earlier, changes in these parameters are part of normal brain development. However, the albumin leakage over the BBB was most pronounced in pups with the lowest bodyweight and was a pathological finding that could not have been related to maturation. Therefore, albeit the limited number of litters, this study may imply that growth restriction, even within the normal range [46], may sensitize the brain to adverse events such as IHH. Indeed, birth weight at term within the normal range correlates with longitudinal brain volume development [47]. Furthermore, being born small-for-gestational-age (SGA) deteriorates neurodevelopmental outcome [48] and delays white matter maturation at term-equivalent age in preterm babies [49]. To explore if an interaction between growth and IHH exists, studies where growth restriction is a controlled condition should be performed.
(II) What is the role of key cellular players in the formation of BBB integrity like pericytes [50] and mediators of angiogenesis like vascular endothelial growth factor (VEGF) [38] in the alterations observed after IHH? Endothelial coverage of pericytes closes the time-window when immature vessels are vulnerable to obliteration by hyperoxia [51], and hypoxia causes the same cells to migrate away from vessels, further destabilizing the endothelium [52]. Furthermore, although angiogenesis in tissue hypoxia is most likely beneficial, its mediator VEGF increases vessel permeability [53]. Future studies should probe whether alterations in pericyte development and VEGF secretion form a causal basis for the observations of increased vascular density, higher T_2_-relaxation time and long-term BBB leakage reported in the present study.

## Conclusion

IHH led to subtle changes in brain white matter diffusivity, grey matter water content and vascular density. However, alterations in BBB permeability may indicate permanent long-term effects. The formation of the neurovascular unit may represent a common site of injury in both the retina and the brain. Furthermore, alterations after IHH exposure were more severe in lower-weight animals, and future studies should aim at exploring whether an interaction between inhibited postnatal growth and IHH exists. 

## Supporting Information

Figure S1 **Regions of Interest in T_2_ and FA-maps.** (a) T_2_-map corresponding to –1.5 mm from the bregma with regions of interest (ROI) marked in putamen (blue). (b) T_2_ -map corresponding to –3.25 mm from the bregma with ROI in parasagittal cortex (green), hippocampus (red) and thalamus (pink). (c) Colour-encoded directional FA-maps from postnatal day 14 (c) and 28 (d) with ROI in corpus callosum (blue), hippocampal fimbriae (green), internal capsule (red) and external capsule (pink). The ROI in corpus callosum encompassed three slices anterior and three posterior to the one shown. The ROI in hippocampal fimbriae, external and internal capsule encompassed two posterior to the one shown.
(EPS)

## References

[1] Msall ME, Phelps DL, DiGaudio KM, Dobson V, Tung B et al. (2000) Severity of neonatal retinopathy of prematurity is predictive of neurodevelopmental functional outcome at age 5.5 years. Pediatrics 106: 998-1005. doi:10.1542/peds.106.5.998. PubMed: 11061766. 11061766

[2] Löfqvist C, Engström E, Sigurdsson J, Hård AL, Niklasson A et al. (2006) Postnatal head growth deficit among premature infants parallels retinopathy of prematurity and insulin-like growth factor-1 deficit. Pediatrics 117: 1930-1938. doi:10.1542/peds.2005-1926. PubMed: 16740833. 16740833

[3] Msall ME (2006) The retina as a window to the brain in vulnerable neonates. Pediatrics 117: 2287-2289. doi:10.1542/peds.2006-0385. PubMed: 16740879. 16740879

[4] Penn JS, Henry MM, Wall PT, Tolman BL (1995) The range of PaO_2_ variation determines the severity of oxygen-induced retinopathy in newborn rats. Invest Ophthalmol Vis Sci 36: 2063-2070. PubMed: 7657545. 7657545

[5] Di Fiore JM, Kaffashi F, Loparo K, Sattar A, Schluchter M et al. (2012) The relationship between patterns of intermittent hypoxia and retinopathy of prematurity in preterm infants. Pediatr Res 72: 606-612. doi:10.1038/pr.2012.132. PubMed: 23037873. 23037873 PMC4433009

[6] Cunningham S, McColm JR, Wade J, Sedowofia K, McIntosh N et al. (2000) A novel model of retinopathy of prematurity simulating preterm oxygen variability in the rat. Invest Ophthalmol Vis Sci 41: 4275-4280. PubMed: 11095626. 11095626

[7] Martin RJ, Wang K, Koroglu O, Di Fiore J, Kc P (2011) Intermittent hypoxic episodes in preterm infants: do they matter? Neonatology 100: 303-310. 21986336 10.1159/000329922 PMC3252018

[8] Schwartz ML, Vaccarino F, Chacon M, Li Yan W, Ment LR et al. (2004) Chronic neonatal hypoxia leads to long term decreases in the volume and cell number of the rat cerebral cortex. Semin Perinatol 28: 379-388. doi:10.1053/j.semperi.2004.10.009. PubMed: 15693394. 15693394

[9] Ment LR, Schwartz M, Makuch RW, Stewart WB (1998) Association of chronic sublethal hypoxia with ventriculomegaly in the developing rat brain. Dev Brain Res 111: 197-203. doi:10.1016/S0165-3806(98)00139-4. PubMed: 9838111. 9838111

[10] Ogunshola OO, Stewart WB, Mihalcik V, Solli T, Madri JA et al. (2000) Neuronal VEGF expression correlates with angiogenesis in postnatal developing rat brain. Dev Brain Res 119: 139-153. doi:10.1016/S0165-3806(99)00125-X. PubMed: 10648880. 10648880

[11] Ramani M, van Groen T, Kadish I, Bulger A, Ambalavanan N (2013) Neurodevelopmental impairment following neonatal hyperoxia in the mouse. Neurobiol Dis 50: 69-75. doi:10.1016/j.nbd.2012.10.005. PubMed: 23064437. 23064437 PMC3534920

[12] Schmitz T, Endesfelder S, Reinert M-C, Klinker F, Müller S et al. (2012) Adolescent hyperactivity and impaired coordination after neonatal hyperoxia. Exp Neurol 235: 374-379. doi:10.1016/j.expneurol.2012.03.002. PubMed: 22449476. 22449476

[13] Chen J, Smith LE (2007) Retinopathy of prematurity. Angiogenesis 10: 133-140. doi:10.1007/s10456-007-9066-0. PubMed: 17332988. 17332988

[14] Hartnett ME, Penn JS (2012) Mechanisms and Management of Retinopathy of Prematurity. N Engl J Med 367: 2515-2526. doi:10.1056/NEJMra1208129. PubMed: 23268666. 23268666 PMC3695731

[15] Vangberg TR, Skranes J, Dale AM, Martinussen M, Brubakk A-M et al. (2006) Changes in white matter diffusion anisotropy in adolescents born prematurely. NeuroImage 32: 1538-1548. doi:10.1016/j.neuroimage.2006.04.230. PubMed: 16843682. 16843682

[16] Martinussen M, Fischl B, Larsson HB, Skranes J, Kulseng S et al. (2005) Cerebral cortex thickness in 15-year-old adolescents with low birth weight measured by an automated MRI-based method. Brain 128: 2588-2596. doi:10.1093/brain/awh610. PubMed: 16123146. 16123146

[17] Khwaja O, Volpe JJ (2008) Pathogenesis of cerebral white matter injury of prematurity. Arch Dis Child Fetal Neonatal Ed 93: F153-F161. PubMed: 18296574. 18296574 10.1136/adc.2006.108837 PMC2569152

[18] Braun A, Xu H, Hu F, Kocherlakota P, Siegel D et al. (2007) Paucity of pericytes in germinal matrix vasculature of premature infants. J Neurosci 27: 12012-12024. doi:10.1523/JNEUROSCI.3281-07.2007. PubMed: 17978043. 17978043 PMC6673365

[19] El-Khoury N, Braun A, Hu F, Pandey M, Nedergaard M et al. (2006) Astrocyte end-feet in germinal matrix, cerebral cortex, and white matter in developing infants. Pediatr Res 59: 673-679. doi:10.1203/01.pdr.0000214975.85311.9c. PubMed: 16627880. 16627880

[20] Roth AM (1977) Retinal vascular development in premature infants. Am J Ophthalmol 84: 636-640. PubMed: 563174. 563174 10.1016/0002-9394(77)90377-4

[21] Wigglesworth JS, Pape KE (1978) An integrated model for haemorrhagic and ischaemic lesions in the newborn brain. Early Hum Dev 2: 179-199. doi:10.1016/0378-3782(78)90010-5. PubMed: 569048. 569048

[22] Yager JY, Ashwal S (2009) Animal models of perinatal hypoxic-ischemic brain damage. Pediatr Neurol 40: 156-167. doi:10.1016/j.pediatrneurol.2008.10.025. PubMed: 19218028. 19218028

[23] Kanaan A, Farahani R, Douglas RM, LaManna JC, Haddad GG (2006) Effect of chronic continuous or intermittent hypoxia and reoxygenation on cerebral capillary density and myelination. Am J Physiol-Reg I 290: R1105-R1114. PubMed: 16322350. 10.1152/ajpregu.00535.2005 16322350

[24] Sirinyan M, Sennlaub F, Dorfman A, Sapieha P, Gobeil F Jr. et al. (2006) Hyperoxic exposure leads to nitrative stress and ensuing microvascular degeneration and diminished brain mass and function in the immature subject. Stroke 37: 2807-2815. doi:10.1161/01.STR.0000245082.19294.ff. PubMed: 17008616. 17008616

[25] Vottier G, Pham H, Pansiot J, Biran V, Gressens P et al. (2011) Deleterious effect of hyperoxia at birth on white matter damage in the newborn rat. Dev Neurosci 33: 261-269. doi:10.1159/000327245. PubMed: 21659719. 21659719

[26] Gerstner B, DeSilva TM, Genz K, Armstrong A, Brehmer F et al. (2008) Hyperoxia causes maturation-dependent cell death in the developing white matter. J Neurosci 28: 1236-1245. doi:10.1523/JNEUROSCI.3213-07.2008. PubMed: 18234901. 18234901 PMC4305399

[27] Coleman RJ, Beharry KDA, Brock RS, Abad-Santos P, Abad-Santos M et al. (2008) Effects of brief, clustered versus dispersed hypoxic episodes on systemic and ocular growth factors in a rat model of oxygen-induced retinopathy. Pediatr Res 64: 50-55. doi:10.1203/PDR.0b013e31817307ac. PubMed: 18344903. 18344903

[28] Paxinos G, Watson C (2008) The rat brain in stereotaxic coordinates. London, UK: Academic Press. 10.1016/0165-0270(80)90021-7 6110810

[29] Saunders NR, Liddelow SA, Dziegielewska KM (2012) Barrier mechanisms in the developing brain. Front Pharmacol 3: 46. 22479246 10.3389/fphar.2012.00046 PMC3314990

[30] Bockhorst KH, Narayana PA, Liu R, Ahobila-Vijjula P, Ramu J et al. (2008) Early postnatal development of rat brain: In vivo diffusion tensor imaging. J Neurosci Res 86: 1520-1528. doi:10.1002/jnr.21607. PubMed: 18189320. 18189320

[31] Mori S, Zhang J (2006) Principles of diffusion tensor imaging and its applications to basic neuroscience research. Neuron 51: 527-539. doi:10.1016/j.neuron.2006.08.012. PubMed: 16950152. 16950152

[32] Hüppi PS, Dubois J (2006) Diffusion tensor imaging of brain development. Semin Fetal Neonatal Med 11: 489-497. doi:10.1016/j.siny.2006.07.006. PubMed: 16962837. 16962837

[33] Morken TS, Widerøe M, Vogt C, Lydersen S, Havnes M et al. (2013) Longitudinal diffusion tensor and manganese-enhanced MRI detect delayed cerebral gray and white matter injury after hypoxia-ischemia and hyperoxia. Pediatr Res 73: 171-179. PubMed: 23174702. 23174702 10.1038/pr.2012.170

[34] Cai J, Tuong CM, Zhang Y, Shields CB, Guo G et al. (2012) Mouse intermittent hypoxia mimicking apnoea of prematurity: effects on myelinogenesis and axonal maturation. J Pathol 226: 495-508. PubMed: 21953180. 21953180 10.1002/path.2980 PMC4524780

[35] Brehmer F, Bendix I, Prager S, van de Looij Y, Reinboth BS et al. (2012) Interaction of inflammation and hyperoxia in a rat model of neonatal white matter damage. PLOS ONE 7: e49023. doi:10.1371/journal.pone.0049023. PubMed: 23155446. 23155446 PMC3498343

[36] Buser JR, Maire J, Riddle A, Gong X, Nguyen T et al. (2012) Arrested preoligodendrocyte maturation contributes to myelination failure in premature infants. Ann Neurol 71: 93-109. doi:10.1002/ana.22627. PubMed: 22275256. 22275256 PMC3270934

[37] Chahboune H, Ment LR, Stewart WB, Rothman DL, Vaccarino FM et al. (2009) Hypoxic injury during neonatal development in murine brain: correlation between in vivo DTI findings and behavioral assessment. Cereb Cortex 19: 2891-2901. doi:10.1093/cercor/bhp068. PubMed: 19380380. 19380380 PMC2774398

[38] Shweiki D, Itin A, Soffer D, Keshet E (1992) Vascular endothelial growth factor induced by hypoxia may mediate hypoxia-initiated angiogenesis. Nature 359: 843-845. doi:10.1038/359843a0. PubMed: 1279431. 1279431

[39] Baburamani AA, Ek CJ, Walker DW, Castillo-Melendez M (2012) Vulnerability of the developing brain to hypoxic-ischemic damage: contribution of the cerebral vasculature to injury and repair? Front - Physiol (Bethesda, Md.) 3: 424 PubMed: 23162470. 10.3389/fphys.2012.00424 PMC3493883 23162470

[40] Pichiule P, LaManna JC (2002) Angiopoietin-2 and rat brain capillary remodeling during adaptation and deadaptation to prolonged mild hypoxia. J Appl Physiol (1985) 93: 1131-1139. PubMed: 12183511. 12183511 10.1152/japplphysiol.00318.2002

[41] Lehmenkühler A, Syková E, Svoboda J, Zilles K, Nicholson C (1993) Extracellular space parameters in the rat neocortex and subcortical white matter during postnatal development determined by diffusion analysis. Neuroscience 55: 339-351. doi:10.1016/0306-4522(93)90503-8. PubMed: 8377929. 8377929

[42] Thornton JS, Amess PN, Penrice J, Chong WK, Wyatt JS et al. (1999) Cerebral tissue water spin-spin relaxation times in human neonates at 2.4 Tesla: Methodology and the effects of maturation. Magn Reson Imaging 17: 1289-1295. doi:10.1016/S0730-725X(99)00063-6. PubMed: 10576714. 10576714

[43] Butt AM, Jones HC, Abbott NJ (1990) Electrical resistance across the blood-brain barrier in anaesthetized rats: a developmental study. J Physiol 429: 47-62. PubMed: 2277354. 2277354 10.1113/jphysiol.1990.sp018243 PMC1181686

[44] Dziegielewska KM, Habgood MD, Møllgård K, Stagaard M, Saunders NR (1991) Species-specific transfer of plasma albumin from blood into different cerebrospinal fluid compartments in the fetal sheep. J Physiol 439: 215-237. PubMed: 1895237. 1895237 10.1113/jphysiol.1991.sp018664 PMC1180106

[45] Chahoud I, Paumgartten FJ (2009) Influence of litter size on the postnatal growth of rat pups: is there a rationale for litter-size standardization in toxicity studies? Environ Res 109: 1021-1027. doi:10.1016/j.envres.2009.07.015. PubMed: 19762015. 19762015

[46] Palmer AK, Ulbrich BC (1997) The cult of culling. Fundam Appl Toxicol 38: 7-22. doi:10.1006/faat.1997.2319. PubMed: 9268602. 9268602

[47] Walhovd KB, Fjell AM, Brown TT, Kuperman JM, Chung Y et al. (2012) Long-term influence of normal variation in neonatal characteristics on human brain development. Proc Natl Acad Sci U S A 109: 20089-20094. doi:10.1073/pnas.1208180109. PubMed: 23169628. 23169628 PMC3523836

[48] Leviton A, Fichorova RN, O'Shea TM, Kuban K, Paneth N et al. (2013) Two-hit model of brain damage in the very preterm newborn: small for gestational age and postnatal systemic inflammation. Pediatr Res 73: 362-370. doi:10.1038/pr.2012.188. PubMed: 23364171. 23364171 PMC3642985

[49] Lepomäki V, Matomäki J, Lapinleimu H, Lehtonen L, Haataja L et al. (2013) Effect of antenatal growth on brain white matter maturation in preterm infants at term using tract-based spatial statistics. Pediatr Radiol 43: 80-85. doi:10.1007/s00247-012-2509-9. PubMed: 23160647. 23160647

[50] Daneman R, Zhou L, Kebede AA, Barres BA (2010) Pericytes are required for blood-brain barrier integrity during embryogenesis. Nature 468: 562-566. doi:10.1038/nature09513. PubMed: 20944625. 20944625 PMC3241506

[51] Benjamin LE, Hemo I, Keshet E (1998) A plasticity window for blood vessel remodelling is defined by pericyte coverage of the preformed endothelial network and is regulated by PDGF-B and VEGF. Development 125: 1591-1598. PubMed: 9521897. 9521897 10.1242/dev.125.9.1591

[52] Gonul E, Duz B, Kahraman S, Kayali H, Kubar A et al. (2002) Early pericyte response to brain hypoxia in cats: an ultrastructural study. Microvasc Res 64: 116-119. doi:10.1006/mvre.2002.2413. PubMed: 12074637. 12074637

[53] Schoch HJ, Fischer S, Marti HH (2002) Hypoxia-induced vascular endothelial growth factor expression causes vascular leakage in the brain. Brain 125: 2549-2557. doi:10.1093/brain/awf257. PubMed: 12390979. 12390979

