# Correction: Brain Development after Neonatal Intermittent Hyperoxia-Hypoxia in the Rat Studied by Longitudinal MRI and Immunohistochemistry

**DOI:** 10.1371/annotation/4709b234-7ad3-4b57-95f9-4bc80a51e9b5

**Published:** 2014-01-10

**Authors:** Tora Sund Morken, Axel Karl Gottfrid Nyman, Ioanna Sandvig, Sverre Helge Torp, Jon Skranes, Pål Erik Goa, Ann-Mari Brubakk, Marius Widerøe

There was an error in the Funding statement. The correct Funding is:

This work was supported by a grant from the Liaison Committee between the Central Norway Region Health Authority and the Norwegian University of Science and Technology (to T.S.M. and I.S.). URL: http://www.ntnu.no/dmf/rad/samorg. The funders had no role in study design, data collection and analysis, decision to publish, or preparation of the manuscript.

